# Retrospective Evaluation of the Effects of Yoga on Negative Symptoms and Quality of Life in Schizophrenia

**DOI:** 10.1192/j.eurpsy.2026.11703

**Published:** 2026-07-31

**Authors:** I. Keleş Altun, E. Kartal, H. B. Bahar

**Affiliations:** 1 Psychiatry, Bursa Yüksek İhtisas Training and Research Hospital, Bursa; 2 Psychiatry, Elbistan State Hospital, Maraş, Türkiye

## Abstract

**Introduction:**

Schizophrenia is a chronic, disabling mental disorder affecting 1% of the population and is mainly manifested by delusions and hallucinations, negative symptoms, disorganized behavior, thoughts, and speech (1). Negative symptoms are related to long-term morbidity and poor functioning in individuals with this disorder. While we can efficiently manage positive symptoms, we have not yet made considerable headway in dealing with negative symptoms (2).

**Objectives:**

Although new studies are conducted on psychotherapies, occupational therapies, and exercise therapies for managing negative symptoms, there is insufficient research on the effectiveness of yoga, a form of exercise that combines the body and mind (3,4). This study retrospectively examined the effect of yoga which added to routine programme in a community mental health center (CMHC) on clinical symptom severity, anxiety symptoms and quality of life, comparing it with treatment as usual (TAU) (exercise, occupational therapy).

**Methods:**

The records of 30 patients diagnosed with schizophrenia who participated in yoga therapy conducted by a certified yoga instructor twice a week for 8 weeks at CMCH, with each session lasting one hour, and 30 schizophrenia patients who continued TAU were retrospectively reviewed. The Positive and Negative Syndrome Scale (PANSS), World Health Organization Quality of Life Scale (WHOQOL-BREF), and Beck Anxiety Inventory scores routinely administered before and after yoga therapy and TAU were compared.

**Results:**

The yoga group had a mean age of **43.57 ± 11.56** and the TAU group **42.30 ± 13.00**, no **statistically significant differences were found** in terms of age, education levels, age of onset, the number of hospitalizations and illness duration (P>0.05). The mean number of sessions in the yoga group was **13.73 ± 6.75**. After 8 weeks, the yoga group showed significant increase in **WHOQOL-BREF domain 3 (social relationships; p = 0.001)** and **domain 4 (environmental health; p < 0.001)**, and decreases in **PANSS-P, PANSS-N, and PANSS-G scores (p < 0.001)**. In contrast, in the TAU group a significant change was observed only in **WHOQOL-BREF domain 1 (physical health;** *p* = 0.014). A covariance analysis of post-test PANSS-N scores showed that (**F(4,55) = 4.73, p = .002, R² = .26**) Pre-test PANSS-N scores significantly predicted post-test outcomes (**F = 18.10, p < .001, η²p = .25**). Participation in the yoga intervention also had a significant effect (**F = 4.75, p = .034, η²p = .08**), indicating greater improvement in negative symptoms compared to the TAU group.

**Conclusions:**

Our results indicate that adding yoga to the rehabilitation program has positive effects on negative symptoms, even when pre-test scores are controlled. Yoga, as both a physical and group-based activity, is thought to contribute to patients’ recovery by promoting social interaction and enhancing quality of life.

**Disclosure of Interest:**

None Declared

